# Cuffless Single-Site Photoplethysmography for Blood Pressure Monitoring

**DOI:** 10.3390/jcm9030723

**Published:** 2020-03-07

**Authors:** Manish Hosanee, Gabriel Chan, Kaylie Welykholowa, Rachel Cooper, Panayiotis A. Kyriacou, Dingchang Zheng, John Allen, Derek Abbott, Carlo Menon, Nigel H. Lovell, Newton Howard, Wee-Shian Chan, Kenneth Lim, Richard Fletcher, Rabab Ward, Mohamed Elgendi

**Affiliations:** 1 Faculty of Medicine, University of British Columbia, Vancouver, BC V6T 1Z3, Canada; manish.hosanee@alumni.ubc.ca (M.H.); gabriel.chan@alumni.ubc.ca (G.C.); kayliewelykholowa@gmail.com (K.W.); rachelcooper@alumni.ubc.ca (R.C.); weeShian.Chan@cw.bc.ca (W.-S.C.); klim@cw.bc.ca (K.L.); 2 Research Centre for Biomedical Engineering, City, University of London, London EC1V 0HB, UK; p.kyriacou@city.ac.uk; 3 Research Center of Intelligent Healthcare, Faculty of Health and Life Science, Coventry University, Coventry CV1 5FB, UK; dingchang.zheng@anglia.ac.uk; 4 Northern Medical Physics and Clinical Engineering, Freeman Hospital, Newcastle upon Tyne NE7 7DN, UK; john.allen@newcastle.ac.uk; 5 School of Electrical and Electronic Engineering, The University of Adelaide, Adelaide, SA 5005, Australia; derek.abbott@adelaide.edu.au; 6 Centre for Biomedical Engineering, The University of Adelaide, Adelaide, SA 5005, Australia; 7 School of Mechatronic Systems Engineering, Simon Fraser University, Burnaby, BC V5A 1S6, Canada; cmenon@sfu.ca; 8 Graduate School of Biomedical Engineering, UNSW Sydney, Sydney, NSW 2052, Australia; n.lovell@unsw.edu.au; 9 Nuffield Department of Surgical Sciences, University of Oxford, Oxford OX3 9DU, UK; newton.howard@nds.ox.ac.uk; 10 D-Lab, Massachusetts Institute of Technology, Cambridge, MA 02139, USA; fletcher@media.mit.edu; 11 Department of Psychiatry, University of Massachusetts Medical School, Worcester, MA 01655, USA; 12 School of Electrical and Computer Engineering, University of British Columbia, Vancouver, BC V6T 1Z4, Canada; rababw@ece.ubc.ca; 13 BC Children’s & Women’s Hospital, Vancouver, BC V6H 3N1, Canada

**Keywords:** photoplethysmogram, photoplethysmography, PPG signal, hypertension assessment, hypertension diagnosis, blood pressure measurement, digital health, digital medicine, wearable technology, wearable devices, pulse oximetry, biomedical engineering, biomedical signal analysis

## Abstract

One in three adults worldwide has hypertension, which is associated with significant morbidity and mortality. Consequently, there is a global demand for continuous and non-invasive blood pressure (BP) measurements that are convenient, easy to use, and more accurate than the currently available methods for detecting hypertension. This could easily be achieved through the integration of single-site photoplethysmography (PPG) readings into wearable devices, although improved reliability and an understanding of BP estimation accuracy are essential. This review paper focuses on understanding the features of PPG associated with BP and examines the development of this technology over the 2010–2019 period in terms of validation, sample size, diversity of subjects, and datasets used. Challenges and opportunities to move single-site PPG forward are also discussed.

## 1. Introduction

Hypertension is a global public health challenge, and its detection, control, and monitoring remain top priorities [1]. Blood pressure (BP) measurement is the principal vital clinical sign used for the detection, diagnosis, and monitoring of cardiovascular and hemodynamic diseases. Traditionally, mercury sphygmomanometers were one of the most common non-invasive approaches to BP measurement [2]. At present, common non-invasive methods of assessing BP include cuff-based auscultatory and automated BP measurements. However, there are considerable disadvantages to these methods. Most notably, cuff-based approaches cannot continuously measure BP, since a 1 to 2 min pause is needed for both cardiodynamic recovery and minimization of measurement errors [3]. Another limitation is that BP measurement is dependent upon adequate inflation of the cuff and compression of the limb. The evidence suggests that continuous BP monitoring is integral to the detection, control, and treatment of hemodynamic diseases, such as hypotension and hypertension [4], but the ability to measure BP in this way is currently limited. Arterial catheterization is a common method of continuously measuring BP, but this approach is invasive, costly, inconvenient, and generally used within an intensive care context. Thus, a novel strategy is critically needed to measure BP both continuously and non-invasively.

### Plethysmography as A Continuous, Non-Invasive Approach to BP Measurement

**Figure 1:**
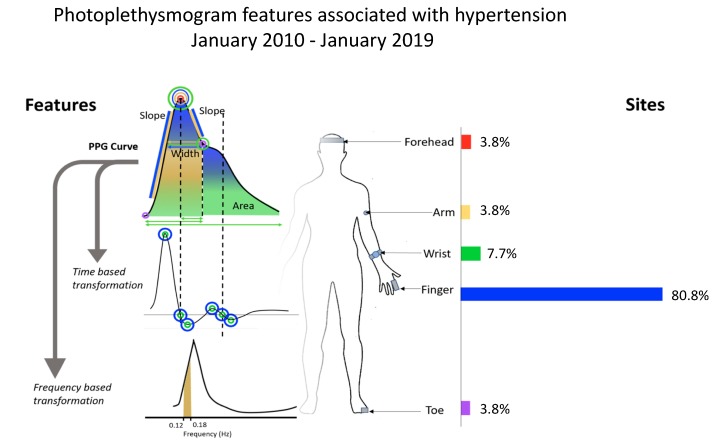
Photoplethysmogram features and measurement sites of single-source photoplethysmography (PPG) in studies conducted between January 2010 and January 2019.

Recently, photoplethysmography (PPG) has been proposed as a continuous, non-invasive approach to BP estimation that can be integrated into wearable devices. PPG records the volumetric pulsations of blood in tissue; these pulsations are associated with the arterial pressure pulse [5]. PPG signals may be integrated with other modalities, such as electrocardiograms, to obtain features such as pulse wave velocity, pulse transit time (PTT), and pulse arrival time (PAT) for BP measurement [6]. There are also features that can be derived from multi-site PPG measurement, as discussed in our previous paper [7]. However, for better applicability in wearable technology, this study focuses on single-source PPG measurements from different anatomical locations. Another approach to estimating BP is the volume-clamp method, in which the device equalizes both external cuff pressure and arterial BP using the Peňáz principle [8]. The amplitude of the PPG signal, which represents blood volume, is frequently compared to a set-point constant, while the cuff pressure is continuously adjusted so that the PPG amplitude is equal to the set point. This allows blood volume in the finger to be maintained by the finger cuff—the pressure of which is presumed to be equal to the systolic BP (SBP). However, this approach still requires a cuff and, as such, may not be adaptable to wearable technology. Figure 1 shows the locations tested in relevant studies conducted between January 2010 and January 2019, as well as multiple non-invasive approaches to estimating BP. Research over the 2010–2019 period has utilized various features of the PPG waveform from a single PPG measurement to improve BP estimation.

Commonly extracted features of the PPG curve include the amplitude, frequency, slope, area under the curve, key points along the PPG curve, and derivatives of the PPG waveform (Figure 1). Although it is possible to integrate PPG into wearable devices, uncertainty remains around the accuracy of this approach. The use of PPG to diagnose hypertension in clinical practice depends on the precision of its BP measurements. A non-invasive, continuous, wearable sensor technology that utilizes PPG for BP measurement could serve as a valuable medical tool worldwide, especially in developing countries where medical interventions are more limited.

This paper reviews the relevant literature published between January 2010 and January 2019 that assesses the reliability of single-site PPG-based approaches. It analyzes the global distribution of these publications, their sample sizes, and the features tested. It also comments on the limitations of various papers and provides recommendations for future investigations.

## 2. Methods

### 2.1. Database and MeSH Terms

**Figure 2:**
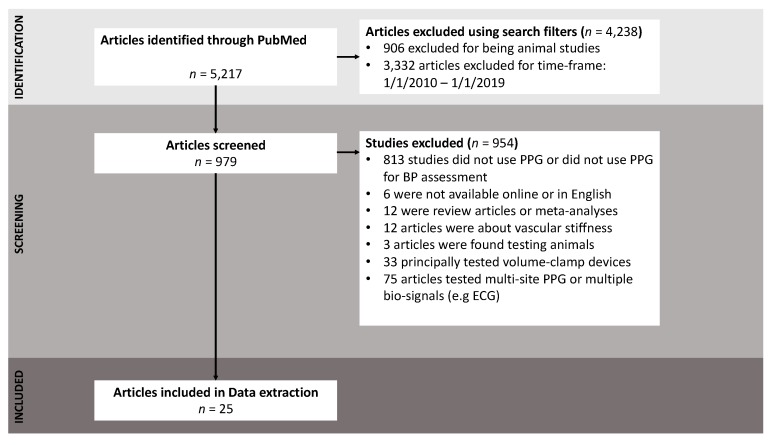
Flow diagram of the exclusion criteria used in this study. From the initial search total (*n* = 5217), 5192 studies were excluded, and 25 studies were identified.

To perform a literature search, we used the PubMed database and filtered search results for the period from January 2010 to January 2019. The search resulted in a total of 25 publications that assessed single-source PPG readings, based on the exclusion criteria shown in Figure 2. The following Medical Subject Headings (MeSH) were used: (((((plethysmography) OR photoplethysmograph) OR photoplethysmography) OR ppg) OR plethysmogram) AND “blood pressure.”

### 2.2. Inclusion and Exclusion Criteria

The goal for the inclusion and exclusion criteria was to focus on studies utilizing a single-site PPG measurement to estimate BP. As shown in Figure 2, 5217 studies were found using the proposed search term. Search filters were used based on publication dates (from 1 Jan 2010 to 1 Jan 2019) and filtered for human studies, resulting in 979 studies. Studies were excluded if they did not use PPG to estimate BP (*n* = 813), if they were review articles or meta-analyses (*n* = 12), if they were unavailable online (*n* = 6), if they tested the volume-clamping technique (*n* = 33), if they tested their approach on animals (*n* = 3), if they used multiple signal types (e.g., ECG, BCG), or if they simultaneously tested multiple anatomical sites for BP estimation (*n* = 74).

## 3. Results

All papers included in this analysis are summarized in Table 1, grouped by anatomical site. As can be seen, the majority of the studies were carried out on the finger. The correlation between extracted PPG feature(s) and the measured SBP varied from 0.35 to 0.82, while the correlation between PPG feature(s) and the measured diastolic blood pressure (DBP) varied from 0.12 to 0.79.

**Figure 3:**
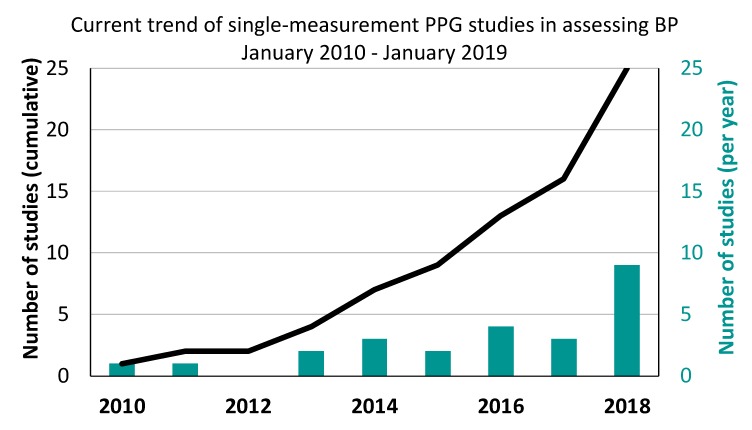
Overall trend of publications that utilized single-measurement PPG to estimate BP from January 2010 to January 2019.

Figure 3 shows the cumulative sum of publications and the total number of studies published by year that utilized single-measurement PPG for assessing BP. The findings indicate that the total number of publications on single-measurement PPG is increasing each year, with the clearest increase occurring in the last few years.

**Figure 4:**
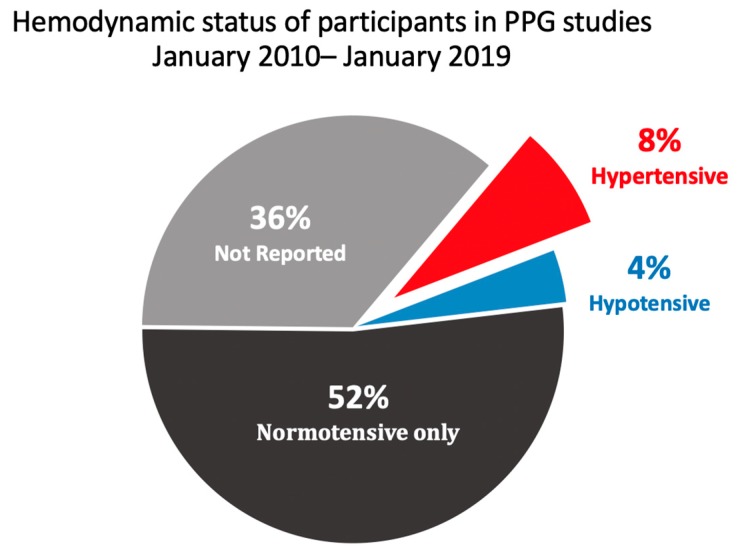
Pie chart of the hemodynamic status of participants in single-measurement PPG studies from January 2010 to January 2019.

Figure 4 shows the number of publications that tested single-site PPG using only normotensive subjects and those that included hypertensive or hypotensive subjects in their sample. The number of studies that did not report the hemodynamic status of their patient sample was classified as “not reported.” Interestingly, 52% of the studies analyzed used only normotensive participants, and 36% of the publications did not report the hemodynamic status of their participant pool. One study tested their approach on a critically ill patient who was hypotensive. Only 8% of all studies tested their approach on a participant cohort that included hypertensive patients.

**Figure 5:**
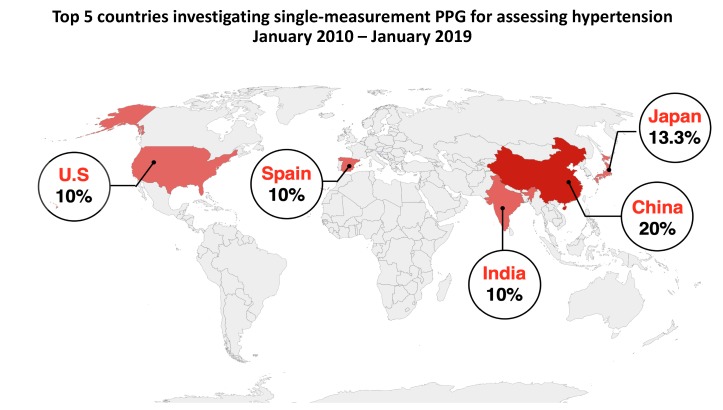
Top 5 countries contributing single-site measurement PPG studies between January 2010 and January 2019. We recommend collaboration between research groups in these countries to identify an optimal approach to BP measurement using single-measurement PPG.

Figure 5 shows the percentage of contributions made by the top 5 countries researching the use of single-measurement PPG to assess BP between January 2010 and January 2019. Papers with multiple authors from different countries were given a score for each country. The country with the most significant contribution was China, contributing approximately 20% of all publications. Japan had the second most publications, with a 13% contribution. The United States of America, Spain, and India also had significant contributions of 10%, respectively.

**Figure 6:**
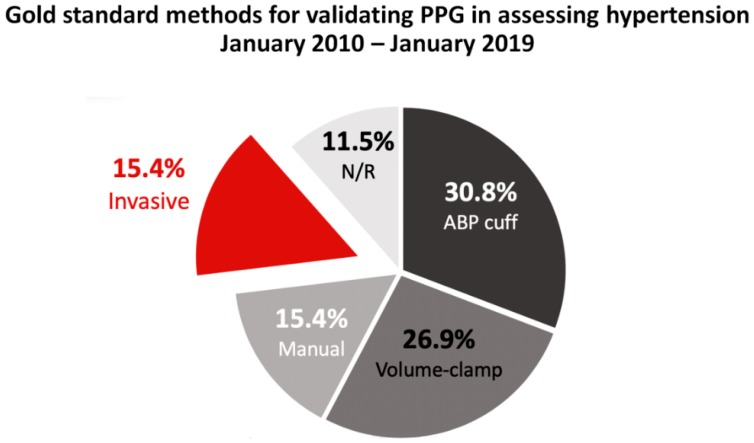
Pie chart of the percentage of studies published between January 2010 and January 2019 that used an automated BP (ABP) cuff, manual BP cuff, or invasive approach as a gold standard. Studies that did not disclose their gold standard method were classified as “not reported” (N/R). Usage of the ABP cuff as the gold standard could be used to assess the validity of PPG in estimating BP from an outpatient setting, while the use of intra-arterial catheters could be used to assess the validity of PPG in an inpatient hospitalized setting (e.g., in critical care units).

Figure 6 shows the specific methods that were used as reference BP measurements in the studies analyzed as percentages. Our findings showed that automated BP cuff-based measurement (ABP) and volume-clamping technique were the most commonly used method for reference BP measurement, observed in 30.8% and 26.9% of all studies, respectively. Manual sphygmomanometer and invasive BP measurement (by intra-arterial catheterization) were used in 15.4% of all studies. There were a considerable number of studies that did not disclose their reference method (11.5%).

## 4. Discussion

### 4.1. Anatomical Site of PPG Measurement

As shown in Figure 2, the number of investigations testing single-measurement PPG for BP estimation has increased exponentially over the 2010–2019 period. The most notable increase in publication volume occurred in 2018, suggesting that single PPG measurement is an emerging topic of research and can be expected to attract more researchers (from academia and industry) in coming years. As the volume of research grows in the near future and more evidence is gathered, single-measurement PPG may offer a promising mechanism for continuous non-invasive BP measurement. However, the total number of identified publications may be limited by the specific MeSH search term used in this study, and the keywords used in each study. Therefore, it is possible that some papers were not identified due to search engine bias, a limitation in the methodological approach, or the use of non-conventional keywords.

The analyzed studies measured PPG signals on the finger, forehead, wrist, or toe, as shown in Figure 1. The most common location was the finger, which was used in 80.8% of all studies. Other anatomical sites were seldom used, such as the wrist, which was used in only 7.7% of studies, followed by the forehead, toe, and arm at 3.8%, respectively. A study by Chua et al. estimated SBP during sleep states by using wrist- or finger-derived PPG and analyzing pulse amplitude, with PAT used as an additional reference [27]. The study found that the correlation between pulse amplitude (using a single-site PPG) and measured SBP was significantly stronger than that for PAT (using multiple biosignals). The results showed that a correlation coefficient for finger PPG feature measurement with BP *R*_S (finger)_ of 0.73, in comparison to the wrist (*R_S_*_(wrist)_ = 0.40) during the non-REM sleep phase. The study also found that pulse amplitude correlation with SBP weakened during REM sleep. This study found that the finger is the anatomical location with the strongest evidence of accurate BP measurement. Therefore, future studies will benefit from finger-derived PPG signals for measurement purposes. More investigations are needed to confirm the validity of other PPG measurement sites.

### 4.2. Integration of Single PPG into Smartphone Applications

Recently, work has been carried out to investigate the efficacy of BP estimation using PPG signals via smartphone cameras or integrated into a portable probe connected to a smartphone. In a 2018 study by Dey et al., single PPG signals were collected from 205 participants (45 for validation, 160 for training) using a Samsung Galaxy S6 heart rate sensor [15]. Using time-domain features, frequency-based features, and patient demographic parameters as features, BP readings were achieved with a mean absolute error of 5 mmHg DBP and 7 mmHg SBP.

Chandrasekhar et al., incorporated PPG and a force sensor unit into a smartphone device to measure BP on a smaller sample size to compare a cuff-based volume-clamp approach and an automated BP device [14]. This group achieved BP readings with a mean absolute error of 9 mmHg SBP and 8 mmHg DBP, along with a correlation coefficient of *R_s_* = 0.76 and *R_D_* = 0.79 with the smartphone, in comparison to *R_s_* = 0.77, *R_D_* = 0.83 with a finger cuff (gold standard). These studies highlight the potential for single-measurement PPG to be used in smartphone-based BP estimations and for the incorporation of this technology into smartphone applications.

Although studies published in 2019 were not included in this paper’s search or review, one notable study by Luo et al. has drawn considerable interest internationally for its study of transdermal optical imaging technology using a smartphone to estimate BP based on facial features [34]. Despite the potential impact of the claims and findings, the study has raised several questions. First, the approach was tested on 1320 normotensive subjects and no hypertensive subjects. Consequently, uncertainty remains as to whether the technology is clinically applicable in diagnosing hypertension. Furthermore, for reasons that are unclear, findings for only 15% of the 1320 subjects were reported. It is also worth bearing in mind that accuracy of measurements may vary in clinical settings due to differences in environmental lighting, camera angle, camera distance, subjects’ skin color, and subjects’ facial features. This paper therefore recommends that further research with a focus on hypertensive patients be carried out.

### 4.3. Varying Study Sample Sizes

Some of the analyzed studies reported large sample sizes. From the 25 published papers that were analyzed, 12 (48%) had more than 30 test participants, with 5 studies having sample sizes of 100 or more. With a sample of 410 normotensive participants (80% used for training), Monte-Moreno et al. tested multiple PPG features of uncalibrated PPG signals using different machine-learning techniques to improve the accuracy of BP estimation. An ML approach called the “random forest technique,” designed by Brienman et al., achieved an *R*_S_ of 0.954 and *R*_D_ of 0.94 between estimated BP and actual BP [26]. In 2013, Ruiz-Rodríguez et al. constructed a regression model with a Deep Belief Network-Restricted Boltzmann Machine (DBN-RBM) to estimate BP among 47 patients (with 525 having been used for training) [24]. These patients were critically ill, but exclusion criteria included arrhythmia, an imminent death condition, and measurement disturbances in the arterial or the PPG curve morphology. The researchers found a large mean bias (MB), where MB_s_ = −3 (±19) mmHg, MB_D_ = −4 (±9) mmHg, and their approach showed promise, but they concluded that the intrinsic variability and the wide agreement limits of their approach did not allow for clinical application [24]. Although these studies reported large sample sizes, both studies used the majority of their participant pool for training rather than for validation of their methods. Future studies could focus on developing an approach that limits the requirement of a large training cohort and thereby maximizes the validation cohort. This paper recommends that future studies continue to use large sample sizes to test their methods.

Two of the 25 analyzed papers utilized only one participant as a sample. One such paper, published by Fu et al.*,* demonstrated qualitatively that a decrease in finger dicrotic wave amplitude correlated with a decrease in SBP readings [22]. Furthermore, they observed a toe inversed dicrotic wave in hypoperfused (low SBP) tissue, suggesting that the dicrotic wave could be used as a sensitive marker for hypoperfusion. In addition, they reported that toe PPG could be more sensitive than finger PPG in measuring BP trends, possibly suggesting that acquisition of hemodynamic information is body-dependent [22]. Another single participant study, by Zahedi et al., was able to estimate the amplitude-normalized waveform of central BP (CBP) [29]. The study used wrist PPG and an autoregression model over a 5-day period on a 25-year-old normotensive male. Such studies have produced significant findings but use of a single participant limits their statistical significance. Future studies could test such approaches on a larger heterogeneous sample size in order to increase the significance of such methods.

### 4.4. Use of Normotensive or Hypertensive Subjects

In order for single-measurement PPG-based technology to be used in clinical assessments of hypertension, sufficient clinical evidence must exist on accuracy among hypertensive patients. As shown in Figure 4, only 8% of analyzed studies conducted their experiment on a participant pool that included hypertensive patients. By contrast, 52% of the studies used normotensive patients only. One notable study by Liang et al. utilized a deep learning Neural Network to automatically identify optimal PPG features for hypertension classification in 121 subjects using the Medical Information Mart for Intensive Care (MIMIC) database [30]. The F1 score, which is an accuracy measure, was 83% when differentiating patients with hypertension and pre-hypertension from those with normotension. However, of those 121 subjects, 97 were used in the training stage, while 24 were used for testing. This study shows promising results in the screening or diagnosis of hypertension, and future studies could build on the findings using a larger cohort of normotensive and hypertensive subjects. The study by Dey et al. also included hypertensive patients in the participant pool [15]. Given the limited number of studies that were conducted on hypertensive subjects, this paper recommends that more studies testing single-measurement PPG on hypertensive patients be conducted in order to advance this field in hypertension diagnosis.

Thirty-six percent of all analyzed studies did not report the hemodynamic status of their participant pool in their manuscripts. This paper observed that the majority of these studies used an online dataset for their participant pool. Notable databases, such as MIMIC or the University of Queensland Vital Signs Dataset, offer a freely accessible pool of relevant patient data (e.g., vital signs, medications, laboratory measurements, diagnostic codes, imaging reports, hospital length of stay, and survival data) for research purposes.

### 4.5. Gold Standard Method of BP Measurement

The use of ABP, intra-arterial catheterization, or volume-clamp methods are justified methods of reference, depending on the researcher’s aims. The analysis showed that only 15.4% of identified studies used the invasive measurement of BP as a reference. By contrast, multiple studies used the ABP (30.8%) and volume-clamp (26.9%) approaches, and manual BP measurement was used only 15.4% of the time. This data makes sense, given that the automated cuff-based approach is routinely used in outpatient clinical settings where hypertension diagnoses can be conducted. Therefore, the usage of ABP as a reference may provide data on how single-measurement PPG compares to the method currently used in clinical outpatient settings to diagnose hypertension. We recommend that future studies continue using ABP as a reference method in order to better assess the applicability of single-measurement PPG in hypertension diagnosis from an outpatient setting.

Invasive arterial BP measurement via intra-arterial catheter offers the advantage of measuring BP continuously and can be used in many inpatient settings [35]. However, as previously discussed, arterial BP measurement has a higher safety risk given its invasiveness and may be less convenient for the patient. Notable complications of intra-arterial BP measurement include vasospasm, occurring in 57% of patients in one study, as well as thrombosis [36]. Such complications indicate a need for a non-invasive continuous approach to BP measurement. The study by Ruiz-Rodríguez et al. used the radial or femoral arteries as the location for invasive arterial reference BP measurement [24]. Acciaroli et al. tested time-series features of PPG integrated into an autoregressive model, measured from the arm, to estimate BP while using radial artery BP measurement as reference. This approach showed promising results, attaining a mean RMSE of 7 mmHg [16]. Given the limited evidence of single-measurement PPG in comparison to invasive approaches, we recommend that more studies be conducted on hospitalized participants who already have an inserted intra-arterial catheter. With this type of investigation, more evidence can be gathered on the use of single-measurement PPG in an inpatient and hospitalized setting.

### 4.6. Studies that Included Pregnant Women

Hypertensive disorders are observed in approximately 5%–10% of all pregnancies, and pre-eclampsia is observed in 3% [37]. Such disorders are associated with higher rates of maternal and fetal mortality and severe morbidity, especially in cases of severe pre-eclampsia and eclampsia [37]. Therefore, it is imperative that such diseases be detected, monitored, and treated. Single-source PPG wearable devices may provide new ways to diagnose and monitor hemodynamic disorders during pregnancy and, as such, could lower maternal or fetal mortality and morbidity. Due to the lack of studies conducted on pregnant individuals, there is a need for more evidence to be gathered within this population.

This paper has concluded that there is limited evidence on the diagnosis of pre-eclampsia using single-measurement PPG. The researchers identified only one paper in which a sample of pregnant women was included. This paper, published by Raichle et al., tested a PPG-based BP estimation app using an algorithm called “Preventicus BP smartphone algorithm,” recorded by an iPhone camera, on 32 pregnant women—one of which was suffering from pre-eclampsia [9]. The application overestimated the BP in women with reference BP in the low range (< 130 mmHg SBP) and underestimated BP in the medium range (130–160). Mean disagreement was 5 ± 14 mmHg, which does not meet the standards of the European Society of Hypertension International as defined in the 2010 protocol revision. However, the authors presented a promising innovation toward health app-based BP estimation via plethysmography. We recommend that future studies conduct experiments that assess the potential for single-measurement PPG in the diagnosis of pre-eclampsia.

### 4.7. Clinical Practice

This paper conducted a review of the literature on single-source PPG, commenting on multiple aspects of these publications, including their sample sizes, the way they defined and tested subjects’ co-morbidities, and the types of gold standards used (if such standards were used at all). It also analyzed the current literature and evidence regarding the use of single-source PPG for hypertensive patients and pregnant women. Wearable devices that use PPG are a promising tool for continuous and non-invasive BP measurement and clinical diagnosis of various hypertensive/hypotensive disorders globally. Integration of this technology into clinical practice could aid in the non-invasive 24 h measurement of BP and may help clinicians to track BP trends among patients, possibly constituting a noteworthy clinical breakthrough that may come to fruition in the next few years. However, some questions remain before it is clinically implemented. One concern is the lack of consensus as to how often PPG should be calibrated. Furthermore, more work needs to be done to identify the optimal feature for BP measurement using single-source PPG. Finally, approaches that use machine learning need to utilize a greater proportion of their sample on device validation, rather than training. Ultimately, single-source PPG-based measurements need to be validated by the Association for the Advancement of Medical Instrumentation, which requires automated sphygmomanometers to have a mean error of ± 5 mmHg and a standard deviation of ± 8 mmHg. Achievement of this level of accuracy must be established prior to clinical implementation [38].

## 5. Conclusions

The findings of this paper indicate that single-measurement PPG is a recent and actively emerging area of research. Analysis of research publications since 2010 provides insights and some recommendations for future studies. This paper recommends the continued use of finger-based PPG measurements and encourages studies to test PPG signals from other anatomical sites. We specifically recommend the continuation of wrist-based studies, which could be useful for application with wearable smartwatch technology. By gathering more evidence on different locations, better comparisons can be made for determining the optimal anatomical location for single-source PPG. The study also strongly recommends that more studies be conducted in hypertensive patients and pregnant women in order to gather evidence regarding the use of such methods for diagnosing hypertensive disorders. Furthermore, future works need to continue collecting ABP and intra-arterial catheterization as gold standards during the collection of PPG signals for validity assessment. Finally, an international collaborative approach between the major contributors is encouraged to ensure scalability and reliability of PPG in assessing hypertension.

## Figures and Tables

**Table 1:** Summary of all identified publications testing single-measurement PPG to estimate BP.

Location	Study	Participants	Age $\left(\bar{mean},\;\mathrm{Range}\right)$	Gold Standard	Signal type (Number of Features)	Error (mmHg)	Correlation Co-Efficient
Finger	Raichle et al. (2018) [9]	32 (all F, pregnant)	$\bar{31.6}$	ABP	PPG (N/R)	MB_s_ = 5 ± 15	R_s_ = 0.401 ^b^
	Hsu et al.(2018) [10]	94 (45:M, 49:F)	$\bar{55}$	N/R	PPG (2)	N/R	R_s_ = 0.354 ^a^
	Alex et al.(2018) [11]	7 (3:M, 4:F)	$\bar{32}$	Volume clamp	PPG (2)	RMSE_S_ < 7	N/R
	Zadi et al.(2018) [12]	15 (8:M, 7:F)	$\bar{28.9}$	Volume clamp	PPG (2)	RMSE_S_ < 8	N/R
	Lin et al.(2018) [13]	22	$\bar{30}$	Volume clamp	PPG (5) + VPG (8) + APG (6)	MB_s_ = 4 ± 9 MB_D_ = 4 ± 5	N/R
	Chandrasekhar et al. (2018) [14]	35	$\bar{39}$	ABP	PPG (4)	MB_s_ = 9 MB_D_ = 8	R_s_ = 0.76 ^b^ R_D_ = 0.79 ^b^
	Dey et al.(2018) [15]	205 (90:M, 115:F)	$\bar{39.8}$	Manual	PPG/VPG/APG (233) + Demographics (3)	MAE_s_ = 7 ± 9 MAE_D_ = 5 ± 6	N/R
	Acciaroli et al. (2018) [16]	8 (7:M, 1:F)	20–40	Invasive	PPG (10)	RMSE = 7 ± 2	N/R
	Shin et al. (2017) [17]	25 (9:M, 16:F)	$\bar{22.5}$	ABP	APG (8) + VPG (4)	N/R	R_s_ = 0.83 ^a^ R_D_ = 0.12 ^a^
	Chen et al. (2017) [18]	10 (5:M, 5:F)	$\bar{24.8}$	Volume clamp	PPG (1)	ME_s_ = −1 ± 4 ME_D_ = 0 ± 3	N/R
	Gao et al. (2016) [19]	65 (40:M, 25:F)	$\bar{29}$	ABP	PPG (22) + Demographics (2)	ME_s_ = 5 ± 4 ME_D_ = 4 ± 4	N/R
	Sun et al. (2016) [20]	19 (14:M, 5:F)	$\bar{28.9}$	Volume clamp	PPG (10) + VPG (4) + APG (4)	RMSE_S_ = 9	R_s_ = 0.85 ^b^
	Suzuki et al. (2015) [21]	50 (20:M, 30:F)	20–70	ABP	VPG (2) + APG (3)	MAE_S_ = 8	N/R
	Fu et al. (2014) [22]	1 (M)	54	ABP	PPG (1)	N/R	N/R
	Kondo et al. (2014) [23]	9 (5:M, 4:F)	$\bar{22.6}$	Manual	PPG (5) + APG (25)	ME_s_ = 6	R_S_ = 0.67 ^b^
	Ruiz-Rodríguez et al. (2013) [24]	572(329:M, 243:F)	$\bar{61}$	Invasive	PPG (N/R)	MB_s_ = −3 ± 19 MB_D_ = −4 ± 9	N/R
	Fukushima et al. (2013) [25]	5 (2:M, 3:F)	$\bar{21}$	Volume clamp	APG (6)	N/R	R = 0.71 ^b^
	Monte-Moreno et al. (2011) [26]	410 (213:M, 197:F)	9–80	Manual	PPG (N/R)	N/R	R_S_ = 0.954 ^b^ R_D_ = 0.94 ^b^
	Chua et al. (2010) [27]	18 (14:M, 4:F)	$\bar{24}$	Volume clamp	PPG (1)	N/R	R_S_ = 0.73 ^a^
Wrist	Atomi et al. (2017) [28]	25	$\bar{22.7}$	Manual	PPG (1) + APG (15) + Demographics (4)	ME_S_ = 2 ± 9	R_s_ = 0.80 ^b^
	Zahedi et al. (2015) [29]	1 (M)	25	ABP	PPG (1)	N/R	N/R
	Chua et al. (2010) [27]	18 (14:M, 4:F)	$\bar{24}$	Volume clamp	PPG (1)	N/R	R_S_ = 0.40 ^a^
Arm	Acciaroli et al. (2018) [16]	8 (7:M, 1:F)	20–40	Invasive	PPG (10)	RMSE = 7 ± 2	N/R
Toe	Fu et al. (2014) [22]	1 (M)	54	ABP	PPG (1)	N/R	N/R
Forehead	Sun et al. (2016) [20]	19 (14:M, 5:F)	$\bar{28.9}$	Volume clamp	PPG (10) + VPG (4) + APG (4)	RMSE_S_ = 9	R_s_ = 0.85 ^b^
N/R	Liang et al. (2018) [30]	121	N/R	Invasive	PPG (N/R)	N/R	N/R
	Duan et al. (2016) [31]	32	N/R	N/R	PPG (15)	MAE_s_ = 5 ± 8 MAE_D_ = 4 ± 6	N/R
	Gaurav et al. (2016) [32]	3000	N/R	Invasive	PPG (12) + APG (23)	ME_s_ = 0.2 ± 7 ME_D_ = 0 ± 5	N/R
	Choudhury et al. (2014) [33]	32	N/R	N/R	PPG (4)	MB_s_ = 1 ± 13 MB_D_ = 1 ± 10	N/R

Note that PPG stands for photoplethysmogram, VPG stands for velocity photoplethysmogram, APG stands for acceleration photoplethysmogram, ABP stands for automated blood pressure measurement, ME_s_ stands for estimated mean error for systolic blood pressure, ME_D_ stands for estimated mean error for diastolic blood pressure, MAE stands for mean absolute error, MB stands for mean bias (calculated using Bland–Altman method), RMSE stands for root mean square error (systolic or diastolic, *R* ^a^ stands for correlation coefficient between PPG feature with measured BP, *R* ^b^ stands for correlation coefficient between estimated BP and measured BP, NN stands for artificial neural network and N/R stands for not reported.

## Abbreviations

*R*	Pearson’s correlation coefficient
MAE	mean absolute error (systolic or diastolic)
MB	mean bias (calculated using Bland–Altman method)
PPG	Photoplethysmogram signal
APG ABP	acceleration PPG automated blood pressure measurement
RMSE	root mean square error (systolic or diastolic)
PE	precision error (systolic or diastolic)
R^a^	correlation coefficient between PPG feature with measured BP
R^b^	correlation coefficient between estimated BP and measured BP
N/R	not reported
